# Interdependent program evaluation: Geographic and social spillovers in COVID-19 closures and reopenings in the United States

**DOI:** 10.1126/sciadv.abe7733

**Published:** 2021-07-28

**Authors:** Michael Zhao, David Holtz, Sinan Aral

**Affiliations:** 1 MIT Initiative on the Digital Economy, 100 Main St., Cambridge, MA 02142, USA.; 2 Haas School of Business, University of California, Berkeley, 2220 Piedmont Ave., Berkeley, CA 94720, USA.; 3 MIT Sloan School of Management, 100 Main St., Cambridge, MA 02142, USA.

## Abstract

In an interconnected world, understanding policy spillovers is essential. We propose a program evaluation framework to measure policy spillover effects and apply that framework to study the governmental responses to COVID-19 in the United States. Our analysis suggests the presence of social spillovers. We estimate that while state closures directly reduced mobility by 3 to 4%, all other states locking down further decreased mobility in the focal state by 8 to 14%. Similarly, while reopening directly increased mobility by 2 to 3%, all other states’ reopening increased mobility in the focal state by 12 to 21%. Our analysis also suggests geographic spillovers: Travel from locked down origins to open destinations increased by 12 to 29%. In contrast, travel from reopened origins to locked down destinations decreased by 6 to 7% for nearby counties and by 14 to 18% for distant counties. Despite its limitations, we believe that our approach takes the first steps toward creating a framework for interdependent program evaluation across policy domains.

## Teaser

COVID-19 policies display significant regional spillover effects necessitating coordination.

## INTRODUCTION

Policy-makers and academics often consider the efficacy of policy interventions in isolation; however, different jurisdictions are connected both geographically and, increasingly, socially. This interconnectedness means that the policies enacted by one governing body affect outcomes beyond the area in which the policy is expected to have a direct effect (*1*). For instance, previous studies have identified the presence of both geographic and social spillovers in contexts such as voting, crime reduction, and physical exercise (*2*–*4*). As the world continues to grow more interconnected, program evaluation methods need to be systematically expanded to assess the effectiveness of policies that exhibit meaningful spillover effects.

Government policy responses to coronavirus disease 2019 (COVID-19) provide a recent and relevant example of the type of policy intervention in which the interdependence between different jurisdictions is important. In the early months of the pandemic, countries around the world imposed strict limits on human mobility by banning gatherings, closing down nonessential businesses, and instituting shelter-in-place orders. Although human mobility levels were already decreasing before the enactment of such policies (*5*, *6*), these measures further reduced the spread of the virus (*5*–*9*) and led to lower morbidity and mortality rates. These policies had effects beyond the counties or states in which they were implemented. These spillover effects need to be explicitly considered, as they can have profound policy implications. For instance, Holtz *et al*. (*10*) found that cross-county spillovers from shelter-in-place policies exceeded the direct impacts of local policies, underscoring the importance of governmental coordination to reduce a potential “loss from anarchy” due to piecemeal implementation of closure policies across regions.

Following the lockdowns, many countries began reopening, allowing the coronavirus room to spread. For example, after reopenings began in the United States, several COVID-19 hot spots emerged, causing some local governments to reimpose social distancing measures. As different regions of the world continue to relax and tighten mobility restrictions, it is critical that we understand how different mobility policy regimes contribute to increases and decreases in COVID-19 case counts. While there is abundant research on the efficacy of shutting down, the body of quantitative research examining the impact of reopening and/or the factors that make reopening safe and effective is still nascent (*11*–*15*). Although most of the research is not causal (*13*, *15*), the more causally rigorous studies (*14*) neglect the magnitude of a policy’s spillover effects relative to its direct effects and do not account for non–travel-related social spillovers. If reopenings cause substantial increases in mobility and exhibit strong spillover effects, then countries that reopen without national coordination could face substantial difficulty in controlling the resurgent spread of the coronavirus and future pandemics. Furthermore, understanding whether the effects of closures and reopenings are symmetric or asymmetric is important in determining the optimal adaptive policy in response to the ebbs and flows of local COVID-19 case rates.

In this work, we combine data from a variety of different sources including the mobility data of more than 22 million mobile devices, daily data on state-level closure and reopening policies, social media connections among 220 million U.S. Facebook users, temperature and precipitation data from 62,000 weather stations, county-level census data, county-level COVID-19 case and death counts, and county- and state-level unemployment. We use these data to measure the direct causal impacts of a state’s COVID-19 closure and reopening policies on its own mobility patterns; the causal spillover effects of other, socially connected states’ closure and reopening policies on a state’s mobility patterns; and the impacts of both origin- and destination-county closure and reopening policies on cross-county mobility, capturing the travel-related spillover effects created by uncoordinated policies implemented across different states and counties. In doing so, we contribute to a growing literature that uses population-scale digital trace data (*16*) to study the impacts of mobility-related interventions in response to COVID-19 and what makes them successful (*5*, *17*). Researchers have shown, for example, that demographic attributes (*18*), political partisanship (*19*, *20*), broadband access (*21*), belief in science (*22*), and information exposure (*23*, *24*) moderate compliance with social distancing policies.

**Fig. 1:**
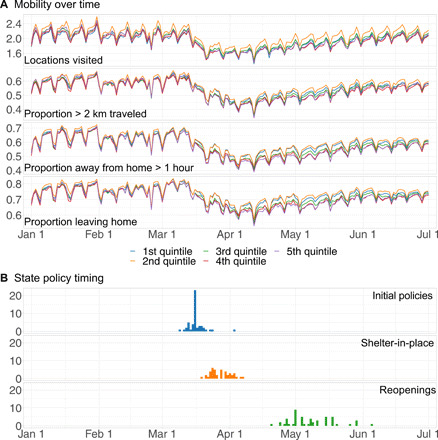
Data on mobility behaviors and state policy timing. (**A**) The time series trends for the number of locations visited per device, the proportion of devices traveling more than 2 km, the proportion of devices spending more than an hour away from home, and the proportion of devices leaving home. Each color represents the averages across clusters of 10 states grouped by how soon they reopened. (**B**) The count of states that enter into a particular policy period on each day.

Our empirical methodology is grounded in a reduced form econometric approach called difference-in-differences (DiD), which is widely used across economics, political science, and public health for policy evaluation. One of the key identifying assumptions of DiD is the “parallel trends” assumption, which states that in the absence of the policy being studied, the time series trends for units that enact the policy (treated units) and those that do not (untreated units) would move in parallel. This assumption is often tested by verifying that the treated and untreated lines move in parallel before the policy intervention in question. Figure 1A shows the time series trends for the four human mobility measures we track, each of which is constructed using data provided by SafeGraph: the daily average number of locations visited by mobile devices, the proportion of devices traveling more than 2 km, the proportion of devices that spend over an hour away from home, and the proportion of devices leaving home. The trend lines for each outcome suggest that in our context, the parallel trends assumption is credible. For DiD to yield unbiased causal estimates, the timing of the treatment also needs to be (conditionally) exogenous. To, as well as is possible, account for potential endogeneity in the timing with which different states enacted different policy interventions and also to make our DiD models robust to deviations from the parallel trends assumption, our DiD models control for weather, COVID-19 case counts, COVID-19 deaths, and unemployment levels using daily temperature and precipitation data from the Global Historical Climatology Network (*25*), daily COVID-19 case and death counts from the *New York Times*, and unemployment data from the U.S. Bureau of Labor Statistics.

Our state-level data on closure and reopening policies come from the COVID-19 U.S. State Policy Database (*26*). During our observation period, different U.S. states implemented a number of different policies, including bans on large gatherings; closures of businesses such as gyms, movie theaters, and restaurants; and shelter-in-place orders with varying degrees of strictness. To create sufficient statistical power to identify causal effects, we collapsed the many different policy interventions into three general classes of policies based on their level of restrictiveness and identify the “periods” during which different states are subject to these policy types. The policy types we analyzed are as follows: “initial policies” (ip), which covers the set of moderately restrictive policies from when a state implements its first closure policy of any kind until it implements a shelter-in-place order; “shelter-in-place” (sh), which covers the more restrictive statewide shelter-in-place orders or stay-at-home orders; and “reopening” (ro), which covers various reopening plans. We also replicated our main results using an alternative middle policy period definition that accounts for a broader set of “movement restriction” policies, as opposed to just shelter-in-place and “stay-at-home” orders (see section S4.3). In all of our analyses, we interpret the effect of the initial policies as a weighted sum of the causal effects of many different mobility-curbing interventions implemented by states across the United States at different points in time, the effect of shelter-in-place as the causal effect of implementing some type of shelter-in-place order, and the effect of reopening as the causal effect of enacting some type of reopening plan. It is worth noting that collapsing COVID-19–related mobility policies into these three broad classes prevents us from measuring the causal effect of any specific policy (e.g., banning indoor dining at restaurants) but enables our causal estimates of direct and indirect policy effects nationwide.

The timing with which different U.S. states entered different policy periods is shown in Fig. 1B. Many states implemented their initial policies in mid-March, during which all four mobility measures were falling (see Fig. 1A). Mobility continued to fall during late March and early April, during which most states implemented some sort of shelter-in-place order. States began enacting reopening plans in late April and continued doing so through early June. During this period, mobility levels rose gradually until they returned to the same levels observed before March.

**Fig. 2:**
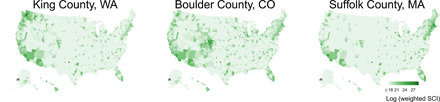
Data on social connectedness. This figure shows examples of the population-weighted SCI used to construct socially weighted measures of alter state policies and behavior.

In addition to measuring the direct effects of a given state’s policies on its own mobility levels, we also measured the effect of other, geographically and socially “connected” states’ policies on each state’s mobility levels. Throughout the paper, we adopt language from the networks literature and refer to the state whose mobility levels we measure as the “ego” state and refer to the states to which it is connected as that state’s “alter” states. To construct the weighted set of counties to which a given county is connected, we combined Facebook’s “Social Connectedness Index” (SCI) (*27*), which uses an anonymized snapshot of the Facebook social network to provide a measure of the intensity of social connectedness between geographic locations, with county-level census population estimates to create a population-weighted SCI. The larger the value of this index for a given ego-alter county pair, the larger the fraction of ego county Facebook friendships that are with people in the alter county. More details on the construction of the population-weighted SCI can be found in sections S1.3 and S1.4, and verification that our results are robust to alternative transformations of the population-weighted SCI can be found in section S4.2. Figure 2 shows choropleth map visualizations of the population-weighted SCI for three different ego counties (King County, WA; Boulder County, CO; and Suffolk County, MA) and each possible alter county. For a given ego county, the alter counties with the highest connectedness index are typically a combination of nearby counties and far away counties with large populations.

**Fig. 3:**
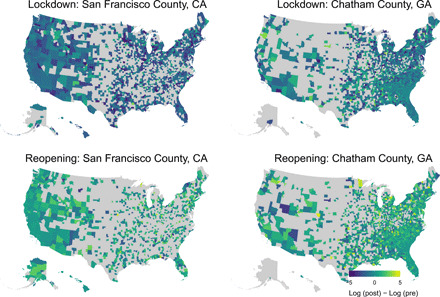
Changes in cross-state mobility patterns after policy changes. This figure shows examples of the difference in travel to a destination county for the 3 weeks before and after a lockdown or reopening.

We also used DiD models to measure the effect of different policies and policy contrasts on cross-state mobility patterns, e.g., how is travel from one state to another affected when one state has begun reopening, whereas the other is still subject to a shelter-in-place order? Figure 3 provides some insight into how policy contrasts can affect cross-county and cross-state mobility. In the weeks following San Francisco County’s lockdown, travel into the county from other counties decreased, whereas in the weeks following the same county’s reopening, inbound travel from other counties increased. The same pattern is also observed for Chatham County, GA.

## RESULTS

**Fig. 4:**
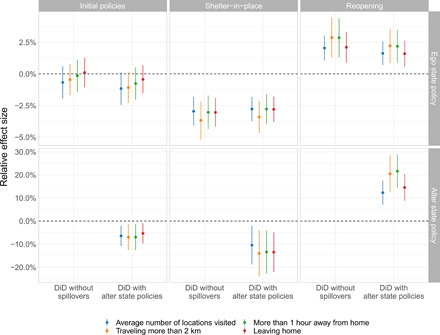
Empirical estimates of the effects of ego state policy and alter state policy on county-level mobility. This figure depicts the estimated impact of both ego and alter state policies. (**Top**) Corresponds to ego state’s policy periods and (**bottom**) corresponds to the alter states’ policy periods. The *x* axis denotes the model specification used to generate the estimates. All estimates are produced using weighted least squares (WLS), with weights determined by county population. SEs are clustered at the ego county U.S. state level.

We first estimated a “no spillovers” DiD model that accounted for the ego state’s mobility-related policies, but not the policies of the ego state’s alter states. The results of that model are found in the top row of Fig. 4. The leftmost column shows that according to the no spillovers model, the combination of initial mobility-related policies deployed across the United States did not cause a statistically significant reduction in mobility, as measured by our four mobility outcomes. In contrast, the middle column of Fig. 4 shows that according to the no spillovers model, statewide shelter-in-place orders reduced mobility within a state by 2.8 to 3.5% on average, depending on the mobility outcome used. Last, the rightmost column shows that according to the no spillovers model, mobility increased by 2.0 to 2.8% on average once a state reopened, depending on the mobility outcome used.

We next estimated a “spillovers” DiD model, in which we accounted not only for the ego state’s policy but also the policies of the ego state’s alter states. In this model, our estimates of the direct effects of each type of policy on human mobility remained quantitatively and qualitatively consistent with the estimates obtained from the no spillovers model (see the top row of Fig. 4). Our estimates of the spillover effects caused by different mobility-related policies are found in the bottom row of Fig. 4. The spillovers model suggests the existence of spillovers caused by social distancing policies, shelter-in-place orders, and reopenings. The leftmost column shows that according to the spillovers model, when the ego county’s alter states began implementing initial social distancing policies, ego county mobility dropped by 6.1 to 8.2%, depending on the mobility outcome used. Furthermore, the middle column shows that according to the spillovers model, when an ego county’s alter states implemented shelter-in-place, ego county mobility dropped by another 8.3 to 11.7%. Last, the rightmost column shows that according to the spillovers model, when an ego county’s alter states began reopening, ego county mobility increased by 12.1 to 21.4%. All of these effects account for the ego counties’ policies and estimate the additional effects of alter states’ policies on ego mobility.

**Fig. 5:**
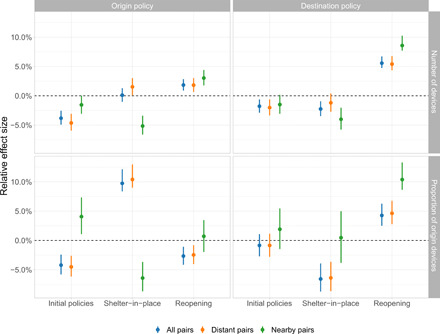
Empirical estimates of origin and destination policies on cross-state travel. In this figure, (**left**) corresponds to the origin policy periods, while (**right**) corresponds destination policy periods (denoted across the *x* axis as initial policies, shelter-in-place, and reopening). (**Top**) reflects policy effects on the number of devices moving from an origin to a destination, estimated using ordinary least squares (OLS), and (**bottom**) reflects policy effects on the proportion of origin devices moving from an origin to a destination, estimated using WLS with weights proportional to origin county population. Colors correspond to estimates produced using all pairs (blue), distant pairs (>100 km) (orange), and nearby pairs (<100 km) (green). Two-way origin and destination state clustered SEs are used to compute 95% confidence intervals.

Figure 5 shows the results from a cross-state travel DiD model that measured the effect of origin- and destination-county policies on travel from the origin county to the destination county. The top row shows the effects on the number of devices traveling from the origin county to the destination county, whereas the bottom row shows the effects on the proportion of devices traveling from the origin county to the destination county. Overall, these results show evidence plausibly consistent with the existence of travel-driven policy spillovers. The right column shows the effects of the destination county implementing different policies. We found that when a destination county was subject to a statewide shelter-in-place order, it received 2.3 to 6.8% less cross-state traffic, depending on the outcome metric used (see blue points). In contrast, being subject to a statewide reopening plan increased cross-state travel to destination counties by 4.2 to 5.4%. In addition to communicating overall effects (blue points), Fig. 5 also communicates the heterogeneous effects of origin- and destination-county policies for distant pairs of counties (orange points) and nearby pairs of counties (green points). We observe notable levels of heterogeneity; for instance, we estimate that destination counties in distant pairs being subject to statewide reopenings increased cross-state travel by 4.5 to 5.3%, whereas destination counties in nearby pairs being subject to statewide reopening plans increased cross-state travel by 8.2 to 9.9%. Insofar as we observe differences in the effects of origin and destination policies across our two outcome variables, we believe that this is due to the fact that the proportion of origin devices traveling to the destination county is bounded between zero and one, whereas the number of devices traveling from the origin county to the destination county is much more highly dispersed. Counties in which there are many origin county devices therefore have much more influence on our “number of devices” estimates.

**Fig. 6:**
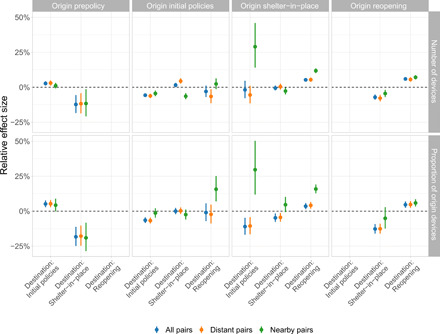
Empirical estimates of origin and destination policy interactions on cross-state travel. Each block in the figure corresponds to different origin policy periods: prepolicy, initial policies, shelter-in-place, and reopening from left to right. (**Top**) reflects policy effects on the number of devices moving from an origin to a destination, estimated using OLS, and (**bottom**) reflects policy effects on the proportion of origin devices moving from an origin to a destination estimated using WLS with weights proportional to origin county population. Different values along the *x* axis of each column correspond to different destination policy periods: destination initial policies, destination shelter-in-place, and destination reopening. Within each column, the marginal effects of each destination policy given the origin policy. Colors correspond to estimates produced using all pairs in blue, distant pairs (>100 km) in orange, and nearby pairs (<100 km) in green. Two-way origin and destination state clustered SEs are used to compute 95% confidence intervals.

Last, when we expanded our cross-state travel DiD model to include all possible origin-destination policy interactions (Fig. 6), several additional findings emerged. The top row of Fig. 6, for example, shows the effects on the number of devices traveling from the origin county to the destination county, whereas the bottom row shows the effects on the proportion of devices traveling from the origin county to the destination county. The second column from the left shows that when origin counties were subject to their own initial policies, the enactment of destination policies did not appear to measurably affect cross-state mobility. However, we estimate that when origin counties were subject to a shelter-in-place order (second column from the right), travel to distant counties that were subject to initial distancing policies decreased by 5.6 to 11.2% (orange points), whereas travel to nearby counties that were subject to initial distancing policies increased by 29.0 to 29.6% (green points). Similarly, we estimate that a destination county reopening while an origin county was still subject to a shelter-in-place order caused cross-state travel from the origin county to the destination county to increase by 11.8 to 15.9% for nearby counties and by 4.2 to 5.4% for distant counties. In contrast, the rightmost column of Fig. 6 shows that origin counties reopening while destination counties were still subject to shelter-in-place appear to have decreased travel from the origin county to the destination county by 4.5 to 5.1% for nearby counties and by 7.8 to 12.6% for distant counties. Last, we estimate that both origin and destination counties reopening caused travel from origin counties to destination counties to increase by 6.8 to 7.1% for distant destination counties, whereas it did not have an effect for nearby destination counties.

## DISCUSSION

Our analysis highlights the importance of taking into account both geographic and social spillovers when evaluating the efficacy of uncoordinated local policy interventions and takes the first steps toward creating a causal framework for measuring the effects of a given policy not only on outcomes in the jurisdiction in which it is enacted but also on outcomes in the jurisdictions to which it is in some way connected. Our study contributes to an emerging literature on the impact of COVID-19 reopening policies (*13*–*15*) and, to our knowledge, is the first to attempt to causally estimate the magnitude of reopening policy spillovers, to explicitly estimate cross-state travel spillovers due to reopening, and to compare estimates of the causal effects of reopening to estimates of the causal effects of related interventions, such as shelter-in-place orders and other policies aimed at curbing human mobility.

That said, this work has a number of limitations. First, there are limitations associated with the data we used to conduct our analyses. Although the SafeGraph panel is sufficiently large to minimize concerns about sampling error, it may exhibit sampling bias as mobile device ownership varies considerably by age and income (*28*). While SafeGraph has shown that their panel is geographically consistent with the U.S. Census population estimates (*29*), it is not clear whether certain demographics are over- or underrepresented, as no device-level demographic data are collected by SafeGraph. Although it is reassuring that we find similar results when using mobility data provided by Facebook as a robustness check (see section S4.1), there are concerns about the representativeness of Facebook’s data as well. It is also possible that mobility datasets from both SafeGraph and Facebook are inaccurate in other, unobservable ways.

Second, there are multiple policy-related questions that are extremely relevant but are beyond the scope of our analysis. For instance, our analysis does not account for the relatively few cases in which local- or county-level policies differed from state-level policies and is restricted to mobility outcomes (i.e., we purposefully avoid extrapolating to health outcomes such as morbidity and mortality rates). We encourage follow-up work on both of these fronts. Accounting for disputes between states and localities will likely enable more accurate assessments of the impact of state-level policies. For example, such disputes may have affected the extent to which state-level policy interventions effectively limited mobility during the COVID-19 pandemic. In addition, while it is widely believed that reductions in mobility drive reductions in new infections and their associated deaths (*30*), rigorously establishing the causal chain from cross-state spillovers to infection rates and deaths is beyond the scope of this paper. Our analysis also does not explore the impacts of specific reopening policies (e.g., resuming restaurant dine-in service or lifting gathering restrictions) and instead measures the average causal effects of different “types” of policies, as defined through “policy periods,” on human mobility.

Third, our analysis may suffer from model misspecification and/or failure to properly quantify the uncertainty associated with our estimates. With regard to model misspecification, our analyses assume that the relationships between the outcomes and independent variables are all linear. Although estimates produced using a “double machine learning” (*31*) estimator (see section S4.7), which can account for nonlinear relationships in the control variables, were nearly the same as those presented in the paper, this method is designed to uncover heterogeneity in the treatment effect estimates themselves, not to test robustness to model misspecification. With regard to the quantification of uncertainty, although our results remain statistically significant when calculating adjacency- and cluster-robust standard errors (SEs) and when conducting Fisherian randomization inference (see sections S4.5 and S4.6), it is possible that there is still correlation structure in the assignment of our treatment variables that we do not properly account for.

Last, although this work represents the initial step toward creating a causal framework for interdependent program evaluation, unambiguous causal identification is likely beyond our reach given the data at hand. To yield causal estimates, DiD relies on both the parallel trends assumption and the assumption that the timing of the treatment is exogenous conditional on observables. Our investigation of potential anticipatory or lagging behaviors (see section S4.4) suggests that the parallel trends assumption is plausible in our setting. However, conditional exogeneity of treatment timing is more difficult to verify. Although our analyses make use of a robust set of controls, it is possible that both our ego policy “treatment” and our alter policy “treatment” suffer from endogeneity. The fact that our ego state policy estimates do not meaningfully change after the inclusion of alter state policies is encouraging, but our estimates of ego state policy effects may yet be confounded by unobserved, time-varying factors that affect both ego state policy enactment and mobility. For instance, COVID-19–related (mis)information shocks may affect both state-level policy and human mobility. Causal interpretation of our alter state policy coefficients presents other challenges, and it is possible that causally interpreting our alter state policy coefficient estimates is an instance of what epidemiologists refer to as the “table 2 fallacy” (*32*), i.e., mistakenly giving causal interpretation to a noncausally identified confounder that is included in a model to account for endogeneity. This could be the case if, for instance, ego-state policies are in part determined by alter state policy and/or mobility levels, or whether both ego state mobility and alter state policy are in part determined by unobserved confounding factors, such as similarities in the demographic composition of connected states. It is also possible that our spillover analysis suffers from what epidemiologists refer to as “the ecological fallacy” (*33*), as we are unable to observe and analyze human mobility outcomes for the specific subsets of people in a given ego county that are socially connected to each possible alter county.

When applied in the context of COVID-19–related mobility interventions, our analysis framework suggests that local social distancing or shelter-in-place orders may only have limited effectiveness and that the totality of the policies implemented by “peers” is approximately three times more effective at reducing mobility compared to the local policy alone. If our causal estimates are accurate, then they indicate that such policy contrasts may create scenarios in which closure policies are counterproductive (*34*, *35*), as those living in locked down regions may travel to reopened regions, potentially causing new COVID-19 hot spots to emerge. Our analysis provides suggestive evidence that such travel spillovers are not only systematic and predictable but also large and, thus, meaningful to our public health. These points are especially important to consider in light of the tiered reimposition of social distancing measures in the United Kingdom or the regional approach taken in California. Our results also suggest that the effects of COVID-19–related mobility interventions are approximately symmetric, i.e., the increase in mobility due to reopenings is roughly equal to the decrease in mobility due to closures. This analysis not only furthers our understanding of the effectiveness of past mobility-related interventions but may also prove helpful in the coming months as local governments may need to reimpose mobility restrictions in response to new COVID-19 hot spots.

Last, beyond human mobility-related interventions, analysis frameworks such as ours may be useful for studying other high-impact policy interventions enacted at the local level, in some cases with little to no coordination. The most pressing example of such policy situations related to COVID-19 is that of vaccine distribution, both within the United States and abroad. State- and country-level variations in vaccine procurement and distribution plans may affect vaccine availability for other states, countries, and regions, as well as the receptivity of a region’s citizens to various distribution plans. Frameworks such as ours could also be applied to non–COVID-related policies such as firearms legislation, policing, criminal law enforcement, legislation governing the labeling and spread of online misinformation, and interventions aimed at combating climate change.

## MATERIALS AND METHODS

### Model specifications

We begin with a basic DiD model that only incorporates each county’s own state policy specified as follows

$$\text{log}(Y_{it})=\delta_{\left(\text{ip}\right)}D_{\text{it}}^{\left(\text{ip}\right)}+\delta_{\left(\text{sh}\right)}D_{\text{it}}^{\left(\text{sh}\right)}+\delta_{\left(\text{ro}\right)}D_{\text{it}}^{\left(\text{ro}\right)}+\theta \prime X_{it}+\alpha_{i}+\tau_{t}+\epsilon_{it},$$ (1)

where log (*Y_it_*) is the log-transformed mobility outcome. The policy variables, $D_{it}^{\left(\text{ip}\right)}$, $D_{it}^{\left(\text{sh}\right)}$, and $D_{it}^{\left(\text{ro}\right)}$ are binary indicators that take the value 1 once county *i* is subject to a statewide closure policy of any sort (ip), a shelter-in-place order (sh), and reopening (ro), respectively. The associated parameters δ_(ip)_, δ_(sh)_, and δ_(ro)_ estimate the average marginal mobility differences for the corresponding policy periods. **X_it_** denotes a set of time-varying control variables—county-level weather, county-level unemployment, county-level morbidity and mortality, state-level unemployment, and state-level morbidity and mortality—while **θ**′ is a vector of the corresponding parameters. Last, α*_i_* and τ*_t_* denote a set of county and time fixed effects and D*_it_* denotes the error term.

We extend this base specification to capture spillover effects with the following specification

$$\begin{array}{cc} \text{log}(Y_{it})= & \delta_{\left(\text{ip}\right)}D_{it}^{\left(\text{ip}\right)}+\delta_{\left(\text{sh}\right)}D_{it}^{\left(\text{sh}\right)}+\delta_{\left(\text{ro}\right)}D_{it}^{\left(\text{ro}\right)}+\gamma_{\left(\text{ip}\right)}D_{-it}^{\left(\text{ip}\right)}+\\ & \gamma_{\left(\text{sh}\right)}D_{-it}^{\left(\text{sh}\right)}+\gamma_{\left(\text{ro}\right)}D_{-\mathrm{it}}^{\left(\text{ro}\right)}+\theta \prime X_{\mathrm{it}}+\alpha_{i}+\tau_{t}+\epsilon_{it} \end{array}$$ (2)

where $D_{-it}^{\left(\text{ip}\right)}$, $D_{-it}^{\left(\text{sh}\right)}$, and $D_{-it}^{\left(\text{ro}\right)}$ denote the weighted average of alter states’ policies, weighted by Facebook connectedness, and where the cross-state policy spillovers are captured by the terms γ_(ip)_, γ_(sh)_, and γ_(ro)_, respectively. For these specifications, we limit our analysis to the 2583 counties with a daily mean device count of at least 500 to minimize measurement error induced by SafeGraph’s differential privacy algorithm. Results presented in the main text cluster SEs at the level of the ego county U.S. state. Our results remain statistically significant when quantifying uncertainty by calculating cluster- and adjacency-robust SEs (see section S4.5).

To measure the impact of policy on cross-county mobility, we use the following specifications

$$\text{log}(Y_{o\to d,t})=\sum_{m}\lambda_{m}D_{ot}^{m}+\sum_{n}\psi_{n}D_{dt}^{n}+\theta_{1}^{\prime }X_{\mathrm{ot}}+\theta_{2}^{\prime }X_{dt}+\alpha_{o\to d}+\tau_{t}+\epsilon_{o\to d,t}$$ (3)

$$\begin{array}{c} \text{log}(Y_{o\to d,t})=\sum_{m}\lambda_{m}D_{ot}^{m}+\sum_{n}\psi_{n}D_{dt}^{n}+\sum_{m}\sum_{n}\pi_{m,n}(D_{ot}^{m}*D_{dt}^{n})+\\ \theta_{1}^{\prime }X_{ot}+\theta_{2}^{\prime }X_{dt}+\alpha_{o\to d}+\tau_{t}+\epsilon_{o\to d,t} \end{array}$$ (4)

Here, log (*Y*_*o* → *d*, *t*_) refers to the log-transformed cross-state mobility from an origin county *o* moving to a destination county in a different state *d* on date *t*. Origin and destination policies are denoted by $D_{ot}^{m}$ and $D_{dt}^{n}$, respectively, where *m*, *n* ∈ {(ip), (sh), (ro)}; **X_ot_** and **X_dt_** denote time-varying origin and destination control variables covering county-level weather, county-level morbidity and mortality, and state-level morbidity and mortality for both the origin and destination. α_*o* → *d*_ and τ*_t_* correspond to directed pair and time fixed effects, and D_*o* → *d*, *t*_ represents the error term. Equation 3 models the impacts of origin and destination policies linearly, whereas Eq. 4 includes all possible interactions between origin and destination policies.

## SUPPLEMENTARY MATERIALS

Supplementary material for this article is available at http://advances.sciencemag.org/cgi/content/full/7/31/eabe7733/DC1
